# The microbiota-gut-brain axis: neurobehavioral correlates, health and sociality

**DOI:** 10.3389/fnint.2013.00070

**Published:** 2013-10-07

**Authors:** Augusto J. Montiel-Castro, Rina M. González-Cervantes, Gabriela Bravo-Ruiseco, Gustavo Pacheco-López

**Affiliations:** ^1^Centro Darwin de Pensamiento Evolucionista and Philosophy Department, Social Sciences and Humanities Division, Universidad Autonoma Metropolitana IztapalapaMexico City, Mexico; ^2^Health Sciences Department, Biological and Health Sciences Division, Universidad Autonoma Metropolitana LermaLerma, Mexico; ^3^Biological Systems Department, Biological and Health Sciences Division, Universidad Autonoma Metropolitana XochimilcoMexico City, Mexico

**Keywords:** microbiota–gut–brain axis, neurobiology, psychoneuroimmunology, evolutionary psychology, social bonds, kissing

## Abstract

Recent data suggest that the human body is not such a neatly self-sufficient island after all. It is more like a super-complex ecosystem containing trillions of bacteria and other microorganisms that inhabit all our surfaces; skin, mouth, sexual organs, and specially intestines. It has recently become evident that such microbiota, specifically within the gut, can greatly influence many physiological parameters, including cognitive functions, such as learning, memory and decision making processes. Human microbiota is a diverse and dynamic ecosystem, which has evolved in a mutualistic relationship with its host. Ontogenetically, it is vertically inoculated from the mother during birth, established during the first year of life and during lifespan, horizontally transferred among relatives, mates or close community members. This micro-ecosystem serves the host by protecting it against pathogens, metabolizing complex lipids and polysaccharides that otherwise would be inaccessible nutrients, neutralizing drugs and carcinogens, modulating intestinal motility, and making visceral perception possible. It is now evident that the bidirectional signaling between the gastrointestinal tract and the brain, mainly through the vagus nerve, the so called “microbiota–gut–vagus–brain axis,” is vital for maintaining homeostasis and it may be also involved in the etiology of several metabolic and mental dysfunctions/disorders. Here we review evidence on the ability of the gut microbiota to communicate with the brain and thus modulate behavior, and also elaborate on the ethological and cultural strategies of human and non-human primates to select, transfer and eliminate microorganisms for selecting the commensal profile.

## INTRODUCTION

There is hardly a place on earth without bacteria. They are found in every habitat imaginable: in every leaf in the lush Amazon forests; below scorching deserts’ sands; within the coldest ice of the Antarctica; and even in the inhospitable environment of the ocean depths, under crushing pressures and in streams of boiling water. Not surprisingly, their realm includes the body surfaces and interior of animals, from minute crawlers to those with exceptional cognitive capacities like the human being. Recent genome sequencing projects suggest that most life forms share up to a third of their genes, and that those in humans show up to a 37% homology with those found in Bacteria and Archaea (McFall-Ngai et al., 2013). The sheer quantity of microorganisms inhabiting human bodies is enormous: more than 1000 different species have been found in a single sample (Relman, 2012). Such data have impacted our self-perception; From a viewpoint of the human body as a self-sufficient individual, to a perception of our bodies as super-complex ecosystems. This change of perspective has included a reappraisal of the role of microorganisms within our bodies (i.e., *endosymbionts*). While the popularly-held belief is that any microorganism found within the human body must have a detrimental effect on its health, emerging research has renewed an emphasis on the fact that many microorganisms have mutually beneficial relationships with their hosts (Archie and Theis, 2011), acting as a *probiotic*: a live microbe with a beneficial effect on the host via modifications of host-associated microbial communities, enhancing the host’s response toward disease, its nutrient-exploitation capacity, or improving its environment (Verschuere et al., 2000). Recent research suggests how *microbiota*, i.e., a microbial community occupying a particular habitat (e.g., the *gut *microbiota), can serve its host by protecting it against pathogens, metabolizing complex lipids and polysaccharides that otherwise would be inaccessible nutrients, neutralizing drugs and carcinogens, modulating intestinal motility, and affecting visceral perception. Across evolution, endosymbionts have established important feedback channels with the central nervous system (CNS), some of which are crucial for maintaining homeostasis. For example, as microbial life was increasingly tolerated across generations of organisms, its presence has shaped the evolution of the immune system (Kelly and Mulder, 2012). The recognition that the gut microbiota influences several signaling pathways led to the suggestion of the concept of a microbiota–gut–brain (MGB) axis, a topic covered by extensive reviews (Rhee et al., 2009; Cryan and Dinan, 2012; Forsythe et al., 2012). The proposal of a MGB axis suggests that through a dynamic alignment, microbiota inhabiting the intestinal lumen affects its host’s CNS activity (including vegetative and cognitive functions), and *vice versa* brain activity impacts microbiota development and composition. Clinical and experimental evidence indicate that this is also the case of human subjects, with such relationship playing a pivotal role in the development of metabolic and mental diseases. According to the World Health Organization, metabolic and mental disorders lead the global burden of disease, urging researchers and clinicians to set research priorities, and to governments, public agencies and private funds to apply urgent actions and investment (Mathers et al., 2008). In this regard, understanding the bidirectional signaling between the microbiota, gut and brain, underlie potential and significant impacts on global health, opening new preventive and therapeutic opportunities. Based on the above, the first section of our work provides an overview of the neurobiology supporting such interactions, focusing on key experimental and clinical data of the MGB axis and its potential impact on relevant metabolic and mental human disorders.

While recent years have witnessed an increasing interest in proximate questions regarding different aspects of microbiota, the coevolutionary interactions of animals and bacteria have been relatively unattended. This, in spite of the possibility that a focus on the evolution of the MGB axis could provide new theoretical frameworks for understanding complex evolutionary relationships involved in mutualisms between hosts and commensal bacteria. This includes the possibility that such mutualisms could influence the evolution of immunological systems, shape higher cognitive functions at the individual level, and work as a selective force promoting socialization and social structures, with an influence on the psychobiological basis of gregariousness, social perception, mate choice, and sexual behavior (Archie and Theis, 2011; Neuberg et al., 2011; Schaller, 2011). Therefore, our review is also aimed at understanding the relationship between the exchange of microbial-life among individuals and sociality. Clear suggestions in this regard have been advanced by Troyer (1984a) and Lombardo (2008). They have championed the hypothesis that social interactions may “tip” the precarious but crucial balance between the costs and benefits of group-living by providing an important but surreptitious benefit in the form of an exchange of mutualistic endosymbiotic microbes (e.g., as a defense against pathogens: Dillon et al., 2005). Thus, the next section, on the relationship between microbial-life and sociality, assumes a comparative viewpoint for evaluating such hypothesis. First, it begins by providing an overview of the evidence of symbioses across different animal taxa. It includes studies that either: (a) focus on the social aspect of endosymbiont-transmission or (b) describe whether an experimental intervention was used to clarify the degree by which the impairment of the vertical or horizontal transmission of endosymbionts may affect an organism’s survival and/or reproductive success. Second, we suggest that the premises of Lombardo (2008) and Troyer (1984a) can be tested by means of the hypothesis that *the similarity of microbial communities across individuals is an index of the strength of their social bonds*. In our opinion, testing this hypothesis may add an important analytical tool to research focused on how social bonds (a social relationship defined by the degree upon which the exchange of any kind of information -or lack of it- has the potential to affect the survival and/or reproductive success of the individuals involved) translate into cooperation and cohesion at the group-level, an approach that could ultimately shed light on the origin and evolution of sociality (Dunbar and Shultz, 2010). We do this via a focus on the association between sociality and direct and indirect means of microbial-transmission in primates, with particular attention to mouth-to-mouth interactions. Finally, by means of an integrative perspective, the last section provides an overview of the different topics covered.

## MICROBIOTA–GUT–BRAIN AXIS AND ITS HEALTH IMPACT

**FIGURE 1:**
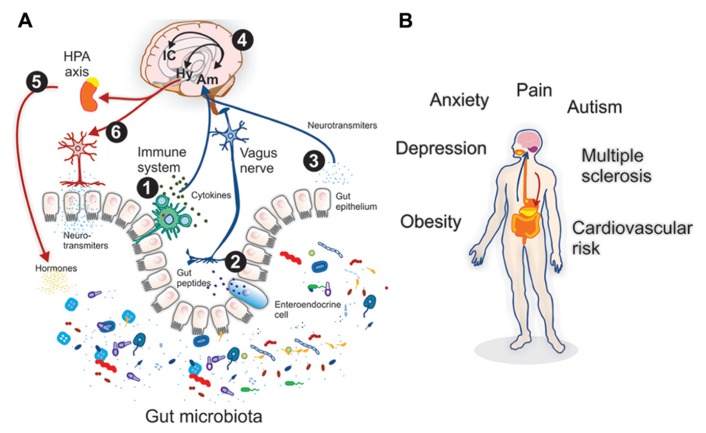
** (A)** Microbiota–gut–brain (MGB) axis. Direct and indirect pathways support the bidirectional interactions between the gut microbiota and the central nervous system (CNS); involving endocrine, immune and neural pathways. On the afferent arm (blue arrows): (1) lymphocytes may sense the gut lumen and internally release cytokines which can have endocrine or paracrine actions, (2) Sensory neuronal terminals, such as on the vagus nerve may be activated by gut peptides released by enteroendocrine cells, (3) Neurotransmitters or its precursors produced as microbiota metabolites may reach the gut epithelium having endocrine or paracrine effects. (4) Centrally, after brainstem relays (e.g., nucleus *tractus solitarii)* a discrete neural network has been described consistently involving the amygdala (Am) and the insular cortex (IC) as main integrators of visceral inputs. Consistently hypothalamic (Hy) activation initiates the efferent arm (red arrows): (5) corticosteroids, release as results of the hypothalamic–pituitary–adrenal (HPA) axis activation, modulates gut microbiota composition. (6) Neuronal efferent activation may include the so called “anti-inflamatory cholinergic reflex” and/or sympathetic activation, both liberating classical neurotransmitters that may affect directly the gut microbiota composition. **(B)** Health conditions affected by the MGB axis. Recent and growing evidence suggests that several health conditions may be affected by intestinal microbiota, including: visceral pain (McKernan et al., 2010; Wang et al., 2010; Clarke et al., 2012), autism spectrum disorders (Adams et al., 2011; de Theije et al., 2011; Thomas et al., 2012; Wang et al., 2012), obesity (Turnbaugh and Gordon, 2009; Davey et al., 2012; Manco, 2012), cardiovascular risk (Tang et al., 2013), anxiety/depression (Bravo et al., 2011; Heijtz et al., 2011; Foster and McVey Neufeld, 2013), and multiple sclerosis (Berer et al., 2011; Lee et al., 2011).

Multiple direct and indirect pathways maintain intensive and extensive bidirectional interactions between the gut microbiota and the CNS; involving endocrine, immune and neural pathways (Grenham et al., 2011), and form the basis of the so called MGB axis (**Figure 1**). For instance, under stress, the brain may influence the composition of the gut microbiota (Bailey and Coe, 1999) via the hypothalamus–pituitary–adrenal (HPA) axis, which regulates cortisol secretion, affecting immune cells activity; both locally in the gut and systemically. When the organism suffers an injury, the first immunological reaction is characterized by redness, pain and heat. These responses are constrained by neuronal regulation of inflammation process, carried out by the HPA axis via catecholamine (Sternberg, 2006) production. The necessary communication processes are based on neurotransmitters, neuropeptides, cytokines, hormones, growth factors (among others), which mediate the relationship between the immune system and the CNS. A feedback process leading to homeostasis (Downing and Miyan, 2000). Yet, disorders like stress (Glaser and Kiecolt-Glaser, 2005)can impact such equilibrium, leading to disease, allergic reactions, inflammatory disease and predisposition to infection. Additionally, cortisol can alter gut permeability and barrier function, and thus contribute to variations in gut microbiota composition (O’Mahony et al., 2011). Vice versa, experimental evidence indicates that the gut microbiota, and pre- and probiotic agents can alter the levels of circulating cytokines, which in turn can have a marked effect on several brain functions (Duerkop et al., 2009; Forsythe and Bienenstock, 2010). Additionally, both the afferent branch of the vagus nerve (Bercik et al., 2011a; Bravo et al., 2011) and modulation of systemic tryptophan, precursor of the neurotransmitter serotonin (Desbonnet et al., 2009), are strongly implicated in relaying the influence of the gut microbiota to the brain.

Experimental approaches on elucidating the MGB axis have so far included, the use of germ-free animals, animals with pathogenic bacterial infections, and animals exposed to probiotic agents or to antibiotics. For instance, germ-free mice have been used to assess neurodevelopmental effects of microbiota loss. Additionally, the administration of probiotic bacteria strains in adult animals or humans has been used to assess the effects of these bacteria on the host. On the other hand, infection studies have been used to assess the effects of pathogenic bacteria on brain and behavior, which are mediated largely through activation of the immune system. Finally, administration of antibiotics can disturb microbiota composition in a temporally controlled and clinically realistic manner and has therefore been a powerful tool to assess the role of the gut microbiota on behavior.

To date, studies investigating the effects of intestinal microbiota composition on brain function predominantly involve animal models of behavioral disorders such as anxiety, depression and cognitive dysfunction; however, accumulating evidence suggests that the composition of the gut microbiota may also have a role in several other metabolic conditions that involve the CNS (**Figure 1**). In addition, recent data have revealed that MGB axis has multiple effects on emotions, motivation and other higher and complex cognitive functions; reviewed elsewhere (Mayer, 2011). Such evidence suggests that various forms of subliminal interoceptive inputs from the gut, including those generated by intestinal microbiota, may even influence memory formation, emotional arousal, affective behaviors and decision making processes (Craig, 2002; Berntson et al., 2003). The human insular cortex and related brain networks (including the anterior cingulate cortex, orbitofrontal cortex and amygdala), have emerged as the most plausible brain regions to support this integration (Craig, 2009).

## MICROBIAL LIFE AND SOCIALITY

### SYMBIOSES ACROSS ANIMAL TAXA

Symbioses have played a substantial role in the development of animal life. In their interaction with the geosphere, they shaped the ancient biosphere in which multicellularity and animal life emerged (Hickman, 2005). Multicellular organisms may have evolved as new conflict-mediation mechanisms (i.e., genetic codes) restricted lower-level individual fitness, increasing that of new individuals at higher levels of biological organization (Michod, 2003). From such humble beginnings and over extended periods of time, a diverse array of cooperative and organized groups of individuals (i.e., societies), have evolved. Since transmission vectors may originate across both the physical and the social environment, microbial communities found in an organism are dependent on its geoecology, physiology, and genotype, but also on the intensity of its social relationships (Archie and Theis, 2011). The following paragraphs provide an overview of selected research describing symbioses between microorganisms and different animal taxa.

Microbial endosymbionts have played an important role in the evolutionary and developmental modification of tissues and organs of several marine or *aquatic* *invertebrates*, for example in the construction of mineralized exoskeletons (Hickman, 2005). Hickman (2005) points out the example of sponges. This group acquires large symbiotic and diverse bacterial communities through vertical transmission, which perform functions like nutrient acquisition, stabilization of the sponge’s skeleton, processing of metabolic waste, and production of important secondary metabolites. The phyla with the largest biomass (e.g., arthropods) are also those with more symbioses reported (McFall-Ngai, 2005). McFall-Ngai (2005) suggests that a crucial difference between vertebrates and invertebrates is based on the relationship between the immune system and its association with microbial life. On the one hand, invertebrates rely on an “innate immune system” consisting of a germline-encoded receptor system associated to cells like macrophages or epithelia. On the other hand, in addition to the innate immune system, vertebrates possess a “combinatorial” immune response (using T-cells and a major histocompatibility complex) which may have evolved as a more “permissive” form of association with long-term endosymbiont microbial consortia, but also as an improved capacity for distinguishing “friend from foe,” leading to the identification of* specific* pathogens (McFall-Ngai, 2005). However, some recent studies suggest that these differences may be less clear-cut than previously thought. For instance, a study in *Daphnia magna* (an aquatic crustacean), found that, compared to those experimentally challenged with a different strain, individuals exposed to the specific strain of the pathogen to which their mothers had been exposed had better fitness, suggesting that some invertebrates may have some kind of specific adaptive immunity (Little et al., 2003). One of the best examples of a beneficial symbiotic-relationship in an aquatic invertebrate is the case of the mutualism between the squid *Euprymna scolopes* and the luminous, symbiotic bacteria *Vibrio fischeri*. This symbiosis is maintained by means of cyclic transmission, where the bacterial symbionts must be acquired from the environment each generation (McFall-Ngai, 1998). *V. fischeri* is first acquired directly from the environment, but then, the light organ of *E. scolopes* undergoes specific metamorphic changes that maintain the symbiosis (McFall-Ngai, 1994). Such relationship helps *E. scolopes* generating bioluminescence, camouflaging it from both prey and predators by eliminating the projection of its shadow (Ruby and McFall-Ngai, 1992). Finally, a study by Verschuere et al. (2000) suggests that *Artemia spp*. is protected from the pathogenic effects of *Vibrio proteolyticus* by specific bacterial strains.

Perhaps the best-studied examples of symbioses and transmission of microbiota is found among social *insects*. The phenomenon received close attention as a model for the study of the origins of sociality, permeating both scientific and popular accounts of the human society during the nineteenth and twentieth centuries (Sleigh, 2002). The niche of the hymenoptera, including a subterranean way of life, large biomass density at the nest, and frequent direct individual contact, can all make them particularly vulnerable to pathogens via a fast-spread of disease among conspecifics (Kaltenpoth and Engl, 2013). Therefore, based on selection at the individual and colony-level, social insects have developed different forms of prophylactic and active responses against some parasite-related costs of social-living, such as: environmental parasite uptake, parasite intrusion (i.e., into a colony), parasite establishment and spread within the colony, and transmission between colonies (Cremer et al., 2007). These responses are referred to as “collective” or “social immunity” by Cremer et al. (2007). Among them is also the possibility for the social-transmission of beneficial microbiota. Recently, Koch and Schmid-Hempel (2011) suggested that both honey and bumble bees present bacterial communities not found among solitary species which, importantly, protect them against a virulent and naturally occurring parasite (*Crithidia bombi*). In an experimental setting, they demonstrate that in order to observe the protective effect of microbiota, individuals had to be exposed to feces from nest mates after pupal eclosion, providing strong evidence for an important benefit of the transmission of microbiota between individuals. On the other hand, Evans and Lopez (2004) suggest that nonpathogenic bacteria may have a positive effect on honey bee immunity, helping them to survive pathogen-infection across different life-stages. In turn, McFrederick et al. (2012) suggested that beneficial *Lactobacillus* found in bees were acquired by both vertical transmission or by contact with flowers, and that the *Lactobacillus* strains associated to Sweet bees could suppress mold-growth and other spoilage organisms at the nest. Other species of eusocial insects feed each other by regurgitation of liquid secretions originating in the crop or alimentary tract (Wilson, 2000), a phenomenon named *trophallaxis* by Wheeler (1918). In termites, trophallaxis allows for the social transmission of protozooans, which they lose after periodic molting but are crucial for the digestion of cellulose (Wilson, 2000).

Experimental studies focused on the transmission of microbiota have been practiced in a few non-eusocial insects. For example, germ-free desert locusts (*Schistocerca gregaria*) were associated with up to three species of locust gut bacteria and then fed with a pathogen (*Serratia marcescens*) by Dillon et al. (2005). Results of this study showed a negative relationship between the density of *Serratia marcescens* and the number of gut bacterial species present, as well as a negative relationship between bacterial community-diversity and the proportion of locusts harboring *Serratia*. A more recent study (Wang and Aksoy, 2012) investigated the role of *Wigglesworthia* *glossinidia* as an endosymbiont associated to the nutrition, fecundity, and development of the immune system in Tsetse flies (Diptera: Glossinidae). The study describes how the peptidoglycan recognition protein (PGRP-LB) is found only in adult flies (as an important component of the milk that nourishes developing progeny), and how the experimental reduction of PGRP-LB decreases female fecundity by damaging the transmission of *Wigglesworthia* through induction of an antimicrobial peptide (Attacin). The conclusion of Wang and Aksoy (2012) is that the transmission of PGRP-LB has a major role in the fitness of Tsetse flies by means of protecting such symbiosis.

There are fewer but equally interesting studies on this subject in fishes, amphibians, and reptiles. Coldwater *fish* appear to acquire their microbiota from the environment after hatching (Hansen and Olafsen, 1999), and there is some indication that different types of probiotic bacteria may have beneficial effects as biological control agents in aquaculture, including immune system improvements (Gómez and Balcázar, 2008) or enhancement of water quality (Verschuere et al., 2000). In the freshwater zebrafish (*Danio rerio*), an experimentally induced lack of microbiota arrests the development of the species’ gut at specific points of differentiation, an effect than can, nevertheless, be reversed by the introduction of bacteria (Bates et al., 2006). In the case of *amphibians*, Walke et al. (2011) found that innate immune defenses with a beneficial effect on the inhibition of the fungal pathogen *Batrachochytrium dendrobatidis* can be vertically transmitted. Their work found that both antimicrobial skin peptides and mutualistic microbiota found in the adult Panamanian “glass-frogs” of the species *Hyalinobatrachium colymbiphyllum*, could be transmitted to embryos, with a possible role of different types of physical contact as a means for this transmission, including female-eggs contact during deposition, and/or male urination on the egg-clutch. Another study by Troyer (1984b) focused on the horizontal acquisition of microbiota in *reptiles*. She investigated how green iguana (*Iguana iguana*) hatchlings employ a significant amount of time in acquiring microbiota before fully exploiting the food resources in their habitat. They do this in a three-step process. First, before having a fully functional digestive activity, they consume soil from within the nest chamber, by which they increase their hindgut microbial populations. Then, up to a week after hatching, they leave the nest and begin eating both plants and soil. Finally, between 2 and 3 weeks after hatching they leave the area around the nest, associate with other conspecifics and eat the feces of older individuals, gaining access to more complex microbial communities.

In comparison, Kohl (2012) presents a thorough review of the many aspects by which microbial communities influence nutrition, development, immunity, and processing of toxins in many species of *birds*. One interesting aspect observed by Kohl is that the symbiotic relationships between birds and microbiota can be, on some occasions, extraordinarily similar to those found in the relationship between mammals and their endosymbionts, while on other instances, they are just slightly distinguishable, by a few, nevertheless remarkable, aspects. A particularly interesting study (Kyle and Kyle, 1993) described by Lombardo (2008) observed that food-provisioning by itself was insufficient for enhancing the survival of orphan chimney swifts nestlings. To achieve this objective, food needed to be coated with the saliva of adults. While all nestlings younger than 6 days receiving food that was not covered by an adult’s saliva died, a high proportion of those that received the saliva-covered food survived.

There is also a vast literature describing the influence of microbiota on a variety of *mammals* (a portion of it reviewed by Lombardo, 2008). Therefore, as a preface for the following subsection, next we focus solely on aspects of the relationship between kinship, lactation and microbiota in human and non-human primates. Several studies have examined the bacterial microbiota of breast-fed and bottle-fed human infants using both conventional plating and molecular techniques. These studies have shown that the large gut microbiota of breast-fed infants is generally dominated by bifidobacteria and lactic acid bacteria, both considered beneficial (Penders et al., 2006). In contrast, the gut microbiota of formula-fed infants is more diverse, but less stable, often containing more *Bacteroides*, *Clostridium* and *Enterobacteriaceae*. Early start of feeding formula milk changes the composition of the intestinal-microbiota, promoting colonization by obligate anaerobes such as the *Clostridium coccoides* group, the *Clostridium leptum* subgroup, *Prevotella* and *Atopobium* cluster during the 3 months after birth (Tsuji et al., 2012). Human milk is a complex bio-fluid containing mainly lactose, lipids, and protein. However, it is not widely recognized that, after lactose and lipids, oligosaccharides are the third largest solid component of human milk. The majority of this type of sugars is not digestible by human infants, instead, their main function may be related to their interaction or support of intestinal microbiota. Oligosaccharides in human milk encourage the growth of beneficial bifidobacteria in the colon, while they also bind competitively to cell adhesion receptors. This binding may prevent pathogen-binding to intestinal epithelial cells and thus pathogenesis. Analysis of the oligosaccharides in human milk resulted in 200 different molecules ranging in size from disaccharides up to approximately 22 residues (Ninonuevo et al., 2006). In this sense, through lactation, mothers provide food for both their infants and the bacteria helping assimilating milk’s nutrients, allowing for the continued inoculation and establishment of infant’s microbiota (Hinde and German, 2012). The importance of this “triangled” relationship is highlighted by the seminal work of Bailey and Coe (1999), who observed that due to strong emotional stress, the disruption of the mother-infant bond (i.e., by separation from the mother) in rhesus monkeys (*Macaca mulatta*) altered the composition of infants’ gut microbiota, increasing their vulnerability to disease. The study of Bailey and Coe (1999) represents an important developmental piece of evidence of a health-related *cost* (i.e., increased vulnerability to disease) observed when the composition of microbiota is altered due to the interruption of microbial-transmission (i.e., by a disruption of the mother-infant bond).

Turnbaugh et al. (2009) found no significant correlations between separation of family members and the degree of similarity between their gut microbiota, as well as no significant differences in the composition of gut microbiota between monozygotic and dizygotic twins, suggesting a likely genetic factor underlying such commonalities. On the other hand, Yatsunenko et al. (2012), characterized bacterial species in fecal samples from 531 individuals from different nationalities. Groups included healthy children and adults from the Venezuelan Amazonas, rural areas of Malawi and US metropolitan areas, and included mono and dizygotic twins. An effect of kinship on gut microbiota was found across countries, focusing the discussion on how differences in social structures may influence the extent of vertical transmission of the microbiota and the flow of microbes among members of a group of people or family. Importantly, their results showed that the phylogeny of fecal microbiota of monozygotic twins was no more similar than the microbiota of dizygotic twins in all age groups tested. Likewise, there were no significant differences in the degree of similarity between the fecal microbiota of mothers and their teenage offspring, nor between teens and their biological fathers. Moreover, the microbiota of co-habiting couples was more similar to each other than to members of other households (Yatsunenko et al., 2012). These results, consistent across the populations studied, suggest that, as in other species, endosymbionts may have an important role in kin-recognition in humans.

### THE ASSOCIATION BETWEEN MICROBIAL TRANSMISSION AND SOCIAL BONDING IN PRIMATES

**FIGURE 2:**
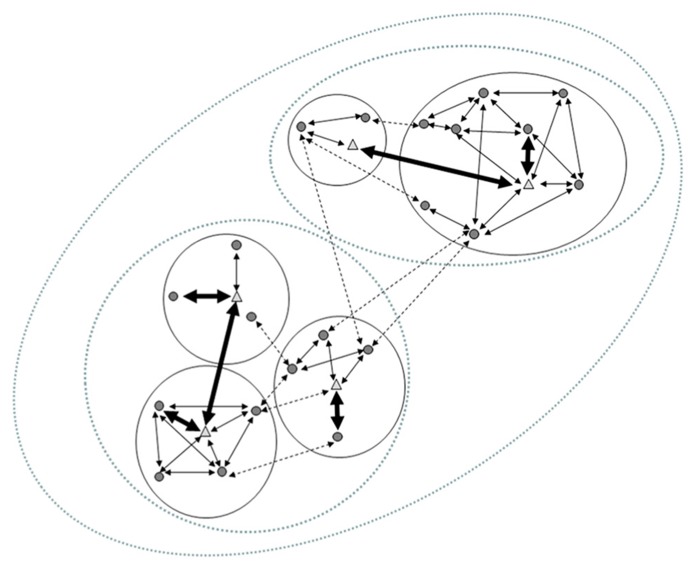
**The typical structure of the multi-layered Hamadryas baboon society.** The smaller social unit (a “one-male-unit”, OMU) in the Hamadryas society is that formed by an adult male (*triangles*), adult females (*circles*) and their offspring. In these units social relationships tend to be circumscribed to members of the same unit (expressed here by arrows, representing social relationships -within bold circles). These units are often formed when larger OMUs fission or when young bachelors sequester peripheral (usually young) females from a large OMU; retaining them in close proximity by force and aggression. Adult females in the same OMU are seldom kin. This produces that strong social relationships (*bold arrows*) are usually established between a female and her unit’s male, not among females in the same OMU. While both sexes can have relatives in other OMUs, social contacts among females from different units are less common (*dotted arrows*), whereas adult males may in fact establish strong alliances with males from other units (which may actually be their kin) forming “clans” (*bold arrows across different* OMUs). Spatial association between individuals of different OMUs (e.g., foraging in relative proximity) can result in the third level of the Hamadryas society: a “band” (*medium-sized dotted ovals*). Finally, different bands congregate in the same cliffs to sleep, forming the largest identifiable group: the “troop” (*largest dotted oval*).

In the case of primates, a behaviorally and cognitively complex social life has evolved across a range of group sizes and social structures as a prerequisite for individual’s development, survival, and reproduction (Mitani et al., 2012). Either in the form of predator deterrence (e.g., Zuberbühler et al., 1997), cooperative breeding (e.g., Burkart and van Shaik, 2010) or group hunting (e.g., Watts and Mitani, 2002), primates use group-level cooperative strategies that in general enhance individual fitness. However, sociality also involves important costs, such as within-group competition for resources (van Schaik and van Noordwijk, 1986) or reduction of reproductive output due to socially induced stress (Dunbar, 1980; Altmann et al., 1988). Thus, a balance between the costs and benefits of social-life is by no means an easy task for primates. By increasing the frequency of contact and proximity between individuals, species living at higher densities, in larger groups, or with promiscuous mating are thought to be the most vulnerable to infection (Altizer et al., 2003). Nonetheless, primates exhibit cohesive and sometimes large groups with strong and long-lasting social bonds (Mitani, 2009) of a kind that, in other animal orders, are almost exclusively found among pair-bonded species (Shultz and Dunbar, 2007). These two observations are somewhat incompatible. However, this apparent paradox could be solved by recognizing the role of partner-choice mechanisms in the structuring of primate societies (Hinde, 1983a). Research suggests that endosymbionts are at the base of different mechanisms for individual recognition and partner selection (Archie and Theis, 2011) underlying social-bonding and the formation of cliques within larger social structures. When partner choice is exerted, not all subjects interact with all other group members, and thus, social bonds (or its absence) may act as a social barrier to pathogen transmission (Loehle, 1995). Hence, if endosymbionts’ transmission is of any benefit to sociality, transmission should be facilitated as social bonds between individuals are stronger, and made more difficult as species use more frequent or more complex partner-selection mechanisms. As partner-choice limits the size of each individual’s social network (Kudo and Dunbar, 2001), the increasing risk of pathogen transmission associated to large groups may set an upper limit to total group size (Freeland, 1976, 1979; Coté and Poulin, 1995; Bonds et al., 2005), leaving the beneficial link between endosymbionts and complex sociality (*sensu* Lombardo, 2008) intact in the form of bonded relationships. For example, the large social groups of the Hamadryas baboon (*Papio hamadryas*: Schreier and Swedell, 2009), classified as a multi-layered, fission-fusion society (Mitani et al., 2012), is a good example of the way social contact is less intense the larger a social-unit is, providing “borders” to microbial-transmission (**Figure 2**). Considering the role of social structure as a barrier against parasite transmission may explain why larger but more subdivided groups, tend to slow the spread of infectious diseases (Griffin and Nunn, 2011; Nunn, 2012), and connect the evolution of the MGB axis to primate sociality. This suggests that the *quality* of social relationships between subjects, but not necessarily the size of their social groups, should be associated to more frequent or direct mechanisms underlying endosymbionts’ transmission between individuals.

#### Indirect mechanisms

In indirect mechanisms of microbial transmission, inoculation is somehow mediated (e.g., by either a free-living pathogen, inanimate environmental features or by another infected host species; Cortez and Weitz, 2013). *Coprophagy* and *urine ingestion* are good examples of indirect mechanisms: often considered abnormal behaviors appearing due to stress (e.g., Baker and Easley, 1996; Nash et al., 1999), they promote beneficial mutualisms required for digestion of plant materials (as in colobus, howler monkeys and gorillas: Milton, 1981; Lambert, 1998; Graczyk and Cranfield, 2003). Even if they exacerbate exposure to parasites, risks can be circumvented by selectively ingesting excretes of relatives while avoiding those of parasitized conspecifics (Lombardo, 2008); two strategies made possible by the widespread individual-recognition capacities of primates (Langergraber, 2012).

Social *traditions* are behaviors maintained and transmitted by social learning that can distinguish between lineages, groups or populations (Avital and Jablonka, 2000). These traditions may represent an important horizontal mechanism for the transfer of microbiota in primates: on the one hand, they can help distinguishing between different chimpanzee communities (Whiten et al., 2001), while on the other, members of contiguous chimpanzee communities can be distinguished based on the contents of their gut microbiota (Degnan et al., 2012). Several social traditions involve the transfer of objects between individuals, such as that observed in *tool-sharing*. For instance, Pruetz and Lindshield (2012) describe how female chimpanzees retrieved tools directly from the mouth of other adults and later used them for their own termite-fishing. In another example, after naíve subjects or infants (both their offspring and other unrelated subjects) approached their tools with their mouths, adult chimpanzees offered them their *previously licked* tools, which were again used and licked (Hirata and Celli, 2003; Hirata, 2006).

Food itself can be another indirect vehicle for microbial transfer. In primates, *food sharing* occurs both among related and non-related individuals (Schaub, 1996; Stevens and Gilby, 2004). However, compared to social insects, food sharing among primates can be a highly selective process of partner choice. In a meta-analysis, Jaeggi and van Schaik (2011) recorded which variables predicted food-sharing among subjects of different primate species. Their results suggested, first, that as greater efforts were required to exploit a particular food-source (i.e., as in tool-use), food sharing between parents and offspring was more common; second, in species with a tendency to share food with infants, sharing between adults was also predicted, although diet-characteristics did not explain food sharing patterns among adults; third, instead, food sharing among *unrelated* adults was predicted by their propensity to exert partner choice, and patterns of *reciprocity* explained interchanges such as “food-for-sex” or “food-for-coalitionary-support.” Particularly relevant for our argument is their observation of an indication that, within single-male species, food sharing between sexes was more common in monogamous species.

#### Direct mechanisms

**FIGURE 3:**
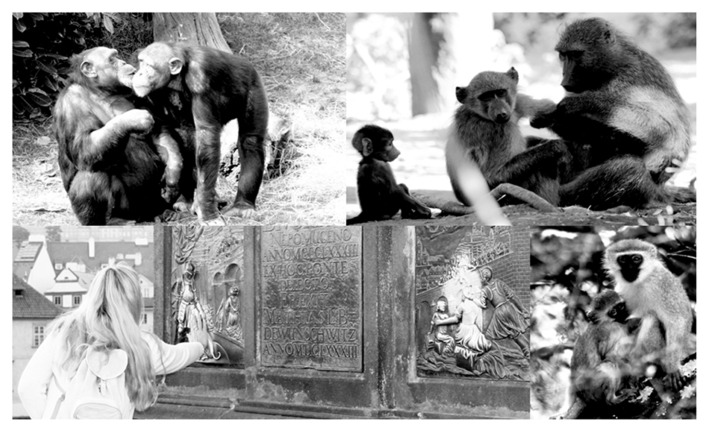
**Behaviors supporting an association between microbial-transmission and social bonding in primates.** The intense sociality of primates provides several different *direct* (i.e., with contact between individuals) and *indirect* (i.e., mediated by any environmental feature) opportunities for the transmission of microbial-life associated to a social-bonding mechanism. Important examples (described in more detail in main text) include: (*upper-left*) mouth-to-mouth contact (in chimpanzees, *Pan troglodytes*): where microbial-life may be directly transmitted in saliva between individuals; (*upper-right*) social grooming (in savannah baboons, *Papio cynocephalus*): where groomers may feed on ectoparasites or food-debris, allowing for the transmission of microbiota; (*bottom-right*) lactation (in vervet monkeys, *Chlorocebus aethiops*): where microbiota directly acquired from the mother helps feeding bacterial communities which in turn help offspring assimilating milk’s nutrients; and (*bottom-left*), indirect transmission of microbiota mediated by a social tradition (i.e., touching religious objects), a possible pathway for the homogenization of microbial-life across individuals of a culturally defined human group (all photos by Augusto J. Montiel-Castro).

Direct mechanisms of transmission of microbial life depend on physical contact between conspecifics (Cortez and Weitz, 2013). Among primates, social* grooming* is the most widespread example (**Figure 3**). During grooming, primates explore their own or other individual’s body surface while removing ectoparasites or debris (e.g., food; Dunbar, 1991), which they often ingest. Primates select their social-partners carefully (Dunbar, 1998). They may exhibit “levels” (Zhou et al., 2005) of acquaintanceship, where those most closely bonded form each other’s immediate “support clique” (Dunbar and Spoors, 1995; Crockford et al., 2008). However, when time available for sociality is scarce or during social instability, they can save social-time by reducing overall sociality, focusing grooming on a few primary partners (Dunbar and Dunbar, 1988; Wittig et al., 2008). Perhaps based on saliva’s healing properties (Gröschl, 2009), different species practice *preening* or *licking* as another form of grooming (Mooring et al., 2004). In primates, this behavior is commonly observed between primate females and their offspring (e.g., apes: Lindburg and Hazell, 1972; *Colobus *sp.: Horwich and Manski, 1975; *Lemur catta*: Nakamichi and Koyama, 2000; *Alouatta palliata*: Duarte-Dias, 2005; *Macaca fuscata*: Turner et al., 2009). While other types of relationship develop through a mutually regulated process of acquaintanceship, as we have described above in detail, the strong bond between primate mothers and their infants is based on important physiological events like lactation (Hinde, 1983b; **Figure 3**).

#### Mouth-to-mouth interactions

Another means of direct microbial exchange is mouth-to-mouth contact (**Figure 3**). While this behavior has been reported for many primates (e.g.,: *Papio anubis*: Bolwig, 1978; *Pan paniscus*: Kuroda, 1980; *Cebus capuchinus*: Manson et al., 1997; *Cebus apella*: de Waal, 2000a;* Pan troglodytes*: Wittig and Boesch, 2003; *Callithrix jacchus:* Kasper et al., 2008; *Homosapiens*: Hughes et al., 2007; *Pongo abelii*: Hardus et al., 2012), different species perform it at different rates and in different contexts. For instance, the possible relationship between mouth-to-mouth exchange of microbiota and affiliative behavior can also be inferred from the *sociosexual* behavior of bonobos (*Pan paniscus*). Compared to that of common chimpanzees (*Pan troglodytes*) bonobo society is less aggressive, more relaxed or friendlier; sex can be used to repair social relationships, and among bonobos but not chimpanzees, reconciliation often involves sexual contact (Wrangham, 1993). Bonobos can mate several times per day; they manipulate other individuals’ genitals with hands or mouth and have more varied forms of copulatory behavior than chimpanzees, including ventro-ventral copulation (i.e., partners facing each other), a sexual position used by bonobo females with their most-trusted partners (Wrangham, 1993). Moreover, bonobos use their tongues intensely (de Waal, 1989), in a way similar to humans, during mouth-to-mouth *kissing*. Being two concomitant forms of “face-to-face” interaction, ventro-ventral copulation and mouth-to-mouth contacts are particularly relevant for supporting the hypothesis that similarities in the composition of microbiota between individuals could be used as indices of bond-strength. This, given that: (1) they allow for the possibility that facial expression may act as a means for the communication of emotional states (Dobson, 2012), while (2) mutual body-contact can stimulate the production of oxytocin, vasopressin or endorphines: neuropeptide mechanisms underlying social-bonding processes in different species (Young and Wang, 2004; Dunbar, 2010); last but not least, (3) during ventro-ventral contact, mouth-to-mouth kissing can act as a means for the *concurrent *reciprocal exchange of microbiota between partners. Altogether, the process could provide the basis for the association of conditioned (e.g., mouth-to-mouth contact) and unconditioned stimuli (e.g., production of oxytocin), reinforcing such behavior through associative learning and thus producing a conditioned response (i.e., oxytocin release) even in the absence of copulation.

#### Kissing: differences across cultures

In humans, mouth-to-mouth kissing is frequently interpreted as the archetypal sign of a strong bond, an index of intimacy and relationship-satisfaction (Gulledge et al., 2003). Eibl-Eibesfeldt (1989, p.138) describes the behavior as follows: “the initiator presses his lips against the partner’s and, when the behavior is fully executed, pushes his tongue between the partner’s lips, while the recipient opens his lips and (in complete execution) begins suckling.” From this archetypal form, some variations can be encountered. While it can be considered a (human) universal sign of affection, there are cultures where, when practiced *in public*, mouth-to-mouth kissing is considered a taboo (Eibl-Eibesfeldt, 1977). Yet, in the opinion of Eibl-Eibesfeldt (1989), as humans are capable of suppressing innate behaviors, this fact does not weaken the argument of its universality. Given that Eibl-Eibesfeldt (1975, 1989) offers an illustrative review of the homogeneity of this behavior across traditional cultures, the focus of the following paragraphs is on aspects of its cross-cultural variation.

Mouth-to-mouth kissing is certainly not found across all human cultures, nor it follows the same behavioral sequence where it is recorded (Eibl-Eibesfeldt, 1975). One occurs in some European traditional villages, where young men chew pine-resin and leave some pieces of it protruding from their mouths. Then, with this resin in their teeth, they playfully “dare” the girl of their romantic-interest to approach and try to pick it with their mouths (Eibl-Eibesfeldt, 1975). Another frequent variation is that observed among groups like Amerindians, Polynesians and Japanese, where, instead of a concurrent touching of lips, people smell (Mykytowycz, 1972) one another. Chamberlain (1906) recorded the observations of D’Enjoy (1897), who suggested that European kisses could be distinguished from Mongolian and Malayan ones given that the latter were forms of “sniffs” and “nose-rubbing,” respectively. Similar observations on human groups from the artic regions (i.e., “Eskimos”), have been interpreted as indications that they sniff each other to check their health: not as a sign of affection, but as a preventive gesture against disease (Washburn Hopkins, 1907). Indeed, the term “Eskimo kiss” is still known and used in English, referring to a “touch of noses” but not of lips. Washburn Hopkins (1907) makes an encyclopedic argument with a detailed analysis of ancient Indian literature, based on which he suggests that ancient Indian words for “kissing” were functionally equivalent to those for “smelling,” and thus that behaviors were similarly used. Another important source of cultural variation of the behavior revolves around who is, and who is not meant to be kissed. In a contemporary and empirical, cross-cultural study of jealousy, women reported being upset when their partner kissed someone else but not when they danced or hugged others, while men reported greater jealousy when their partners had sexual fantasies about other people, compared to when they hugged or danced with others (Buunk and Hupka, 1987). In a more recent study, significant ethnic differences were found among data on the age at which teenagers of different ethnic groups kissed for the first time. Compared to Asian Americans, more African Americans, Caucasians, and Latino/Hispanic subjects had kissed for the first time at earlier ages (Regan et al., 2004). Another cross-cultural study found no differences in the way Asians, versus American students expressed love: intimate behavior, including kissing, was an indicator of love in marriages but not in friendships (Kline et al., 2008). For Frijhoff (1991), regardless of intense historical and contemporary western influence, and the fact that the gesture *is* observed in these cultures, Africans and Asians consider the public performance of mouth-to-mouth kissing as disgusting or immoral, relegating it to the sphere of *intimacy*; not because the gesture is absent, but because it is not recognized as a “legitimate public rite.” It is in these cultures, Frijhoff (1991) suggests, where greetings commonly involve the use of the “sniff-kiss” or/and a bow of the body, or a hand gesture. Chamberlain (1906) and Frijhoff (1991) also refer to contemporary cultural differences related to the greeting aspect of kissing: common (even among males) in Russia or France (where this gesture’s underlying degree of affection may be indexed by its loudness), but rarer in England, the Netherlands or the USA.

The socio-religious aspect of kissing has been thoroughly analyzed by Frijhoff (1991), who makes interesting conclusions distinguishing the public and private aspects of kissing. On the one hand, he suggests, rituals may have adapted kissing and/or embracing as signals of group-membership, a sign of association and/or fraternity, ruled by cultural standards of public expression that provide a sense of group-identity (e.g., the rite of publicly kissing the feet of the statue of St. Peter in the Vatican, or touching a religious icon: **Figure 3**). On the other hand, Frijhoff (1991) suggests that, in the private sphere, both the experience of a religious person (e.g., when kissing a religious icon), and that of lovers during mouth-to-mouth kissing, appear to separate them from the group, creating a sense of psychological intimacy; a likely reason for why, in some cultures, kissing (i.e., mouth-to-mouth) in public is not considered polite.

#### Kissing: evolutionary perspective

In view of the previous analysis, it is somewhat surprising that while kissing is found across several cultures and is a topic of strong popular concern (Walter, 2008), its evolution is not yet fully understood. Hypotheses have certainly been suggested. For example, kissing is one of the many different behaviors observed during reconciliation in non-human primates (de Waal, 1989) suggesting its role as a means for appeasement. However, in spite of substantial evidence highlighting the role of kissing in the context of reconciliation (de Waal, 1989, 2000b), one could ask: if grooming can itself produce intrinsic positive reinforcement (i.e., beta-endorphins) in a primate’s nervous system (Keverne et al., 1989), why would an additional behavior, one increasing the probability of pathogen-transmission, be used during reconciliation? This is an important consideration. Mouth-to-mouth contact and direct exchange of saliva expose individuals to pathogen transmission. In view of this probable cost and its role as an index of reconciliatory tendencies, one would expect it to provide some intrinsic benefit (Hendrie and Brewer, 2010) and to be a highly selective form of inter-individual interaction. Comparative evidence of mouth-to-mouth feeding in parent-offspring dyads across birds and mammals suggests that kissing could evolve as a form of mouth-to-mouth food exchange between offspring and progenitors (Eibl-Eibesfeldt, 1975). However, common marmosets (*Callithrix jacchus*: a new world monkey that exchanges food often) do not restrict these interactions to matting partners or offspring, and tolerate mouth-to-mouth exchanges with both dominants and subordinates (Kasper et al., 2008). Instead, since tolerating or rejecting a stressful event such as the transgression of personal space may provide information about the quality of a relationship, Kasper et al. (2008) suggest that these “up-close” exchanges may serve as tests of the quality of a relationship. For Nicholson (1984), kissing involves some form of social-bonding by means of semiochemical addiction: a direct and continued exchange of sebum and pheromones facilitating bonding and love. The possibility that chemo-signals have a role in communication, via body-secretions has been recently confirmed in humans by de Groot et al. (2012), observing that chemo-signals of fear and disgust can produce multimodal emotional synchronization between sender and receiver, and thus, that communication of emotional states is not restricted to language and visual stimuli. However, results showing that women prefer the scent of males’ t-shirts with whom they have greater immunological dissimilarity (Wedekind et al., 1995), highlight the role of smell (Penn and Potts, 1998) as an alternative route for the development of a purported semiochemical-addiction.

The research of Hughes et al. (2007) on the relationship between pair bonding strategies and kissing in humans, points out other important aspects of this behavior. While men may use kissing for increasing the likelihood of sexual intercourse, women use it as a form of mate-assessment and a behavioral-monitor of the quality of long-term relationships (Hughes et al., 2007). This affiliative aspect of kissing may also be interpreted as a willingness to sustain close social bonds *at the risk of* contracting an illness (Hughes et al., 2007). Therefore, kissing has also been suggested as a strategy aimed at avoiding contagion by pathogens such as the human cytomegalovirus during infant’s gestation, for which testing for such possibility before conception or before the onset of vulnerable periods of fetal development would be highly advantageous (Hendrie and Brewer, 2010). Moreover, since couples sharing previously used, food-related items “contaminated” by their partner (e.g., a licked spoon) are perceived as “more intimate” by third-parties (Alley, 2012), kissing may also function as a group-oriented “advert” or proxy of the strength of the bond between two individuals.

## FINAL COMMENTS: INTEGRATIVE PERSPECTIVES

### THE MICROBIOTA–GUT–BRAIN AXIS AND ITS IMPACT ON HEALTH

The excitement of emotion, the state of alertness and enhanced activation linking the viscera, in particular heart and gut, to the human mind, as well as the mechanisms for bidirectional signaling between these organs, was among the topics by which Charles Darwin himself advocated evolutionary continuity in *The Expression of Emotions in Man and Animals* (Darwin, 1872). With similar intentions, the present review has suggested how mutualistic endosymbionts may have a crucial role in these processes. Thus, the first integrating ideas emerging from our review are focused on the relevance of the different communication channels between the gut microbiota and the brain. For instance, the crucial relationship described in sections above, that between microbiota, cytokines, short-chain fatty acids, systemic tryptophan and their effect on brain function, signals interesting possibilities for fruitful research. For example, the recognition of the role of microbiota in the modulation of tryptophan and thus serotonin, could complement insights on the social and evolutionary basis of schizophrenia (Burns, 2004), while other fertile approaches should focus on the importance of probiotics as modifiers of health, behavior and mood. Such attempts should provide alternatives in clinical settings, and preventive aspects of some of the most prevalent mental and metabolic disorders of modern human societies, such as depression and obesity.

The reports on the role of gut microbiota as an influence in the formation of memories and emotional arousal suggest the existence of a crucial relationship between interoceptive stimuli and the evolution of higher cognitive processes, one that may be based on a system supporting *empathy*, or a capacity for understanding the feelings of other individuals. On the one hand, results showing how the anterior insular cortex can be activated by the images of other humans experiencing disgust (Wicker et al., 2003) suggest the action of a mechanism homologous to *mirror neurons* (Gallese, 1998). These neurons, first located in the ventral premotor cortex of macaque brains, activate both when subjects perform a particular action and when they observe similar actions performed by other individuals (Rizzolatti and Fogassi, 2007). Mirror neurons may be crucial for understanding the underlying intentions of actions, which has led some to suggest that they are part of the system upon which empathy is constructed (Ferrari et al., 2003). On the other hand, evidence suggests that another kind of cells, “*Von economo*” neurons or “spindle cells” in layer five of the anterior cingulate cortex are particularly involved in processes of self-experience, empathy and social bonding (Parr et al., 2005). For Parr et al. (2005), there are at least two important characteristics suggesting the role of such neurons as a neurological basis of sociality. First, these cells have been identified in humans and apes but not in monkeys, suggesting a recent evolutionary origin associated to higher cognition; second, since they seem to be reduced and abnormally located in autistic individuals, they may underlie the lack of empathy characterizing autism. Finally, such system of representation of emotions of others, may be complemented by the action of the vagus (i.e., the “pneumogastric” nerve, according to Darwin, 1872), as a means for activating responses and control of the metabolic output necessary for social interaction. In this regard, the Polyvagal theory (Porges, 2003) suggests that the myelienated branch of the vagus, found only in mammals, is a key for understanding the non-endocrine bases of social behavior. Given that this branch of the parasympathetic system controls facial expression, swallowing, breathing and vocalizing, and has an inhibitory effect upon the sympathetic system innerving the heart, it promotes the calmness and autonomic substrate of effective social interaction (Porges, 1997). Nonetheless, several details regarding such a system remain to be determined. For example, whether intuitive decision making is based on an interoceptive map of gut responses enabling the brain to make gut-based decisions based on interoceptive stimuli (Preuschoff et al., 2008).

### MICROBIAL LIFE AND SOCIALITY

Lombardo (2008) has provided suggestions as to how to distinguish between the transmission of microorganisms as a causal benefit of social interactions versus a mere correlate, byproduct or cost of sociality. His first suggestion, primarily revised in the first section of this review, involves using antibiotics to modify microbial communities in an organism and then observing the effects of such intervention. Evidence across our review suggests that type of interventions can result in significant fitness effects in the experimental subjects. The second set of tests suggested by Lombardo (2008) are those impairing group-living individuals from horizontal transmission of microbial life but not from social contact. He suggests that by means of such intervention we would gain knowledge on how hosts may fail to thrive, not because of lack of social contact *per se*, but because of the impairment of endosymbiont-acquisition from conspecifics. In this sense, our review of the variety of symbioses found across different animal taxa was aimed at providing evidence describing the varied benefits due to such symbioses, and thus suggesting how an interruption of either vertical or horizontal transmission of microbial life could result in significant costs in terms of fitness. Then, based on evidence supporting the idea that “social immunity” is found among several animal taxa, the relationship between intense sociality and the exchange of microbiota was approached by examining the association between direct and indirect patterns of transmission of microbial-life and the intensity of social-partner selection in primates. For this purpose, we suggest that the hypothesis that more similar microbial communities would be found among subjects with stronger social bonds could be used for testing whether primates also obtain benefits associated to microbial transmission. Ultimately, as Dunbar and Shultz (2010) suggest, adding another operational index of sociality to previous, more orthodox, measures of bondedness (e.g., grooming or inter-individual distances), could help expanding our understanding of the evolution social complexity. Thus, we argue that, if endosymbionts’ transmission is of any benefit to primate sociality, direct transmission of microbial life should be associated to stronger social bonds, but not to large group sizes. Such possibility, we suggest, allows for the beneficial exchange of endosymbionts across individuals, while at the same time, permits the necessary partner-choice mechanisms (associated to social-bonding processes) restricting group-size, leading to the formation of social-borders which limit the extent of microbial transmission. Indeed, research on primates suggests that greater modularity or greater structuring of social groups reduces parasite success (Griffin and Nunn, 2011). Moreover, indirect mechanisms of transmission allow subjects to exert at least three strategies for selection of the microbial load transmitted: first, one focused on the conditions or characteristics of the objects exchanged (i.e., the spoilage of food) allows subjects to decide whether a particular item deserves further processing or not (Laska et al., 2007); second, subjects may directly assess the phenotypical-characteristics of the interacting subjects, deciding whether to engage in social interaction or not. For example, by stressing immigrants in order to “test” whether they carry pathogens before allowing their full integration into a group (Freeland, 1976); third, certain species may apply *a posteriori* mechanisms for the elimination of pathogens like zoopharmacognosy (Huffman, 1997). In contrast, direct interaction restricts those mechanisms to the second and third strategies. Hence, individuals with stronger social bonds incur greater risks when interacting with their close associates compared to cases when they interact with “mere acquaintances,” likely leading to an easier transmission of microbial life among strongly bonded subjects. In this sense, another suggestion stated above was that the transmission of microbial life would be increasingly difficult as primates would employ more complex partner selection mechanisms. From this perspective, since the Social Brain Hypothesis (Dunbar, 1998) is focused on the evolution of both sociality and primate neocortex by means of intense partner-selection due to increasing cognitive capacities, we can relate this hypothesis to the ideas of both Troyer (1984a) and Lombardo (2008) and hypothesize that, in primates: (i) other measures of the strength of social bonds will be positively correlated to the similarity of microbial species shared by members of a social group or reproductive pair; (ii) the number of microbial species shared by any two members of a social group will be positively correlated to that species relative neocortex size; (iii) the number of microbial species common to any two members of a social group and group size will be negatively correlated.

A particular point of concern emerging from this section is the apparent opposition of reports describing the relationship between microbiota and kinship in humans and apes. While both genetically and socially related humans show similar compositions of their gut microbiota (Degnan et al., 2012), the chimpanzees of Gombe showed similarities within sexes, as well as between members of different communities but not between individuals of the same community. This is remarkable, especially when taking into account evidence suggesting that primate females transmit necessary microbiota to their offspring. Such opposing results could imply that, while similarities in microbiota could be a useful index of the strength of social bonds for humans, the index would not be and adequate measure of prosocial tendencies in chimpanzees. In turn, this possibility would reduce the usefulness of the similarity of gut microbiota as a comparative index for examining the strength of social bonds in other species. However, these variations may be explained by pointing out important differences between the social behavior of apes and humans. Despite the fact that the social system of both chimpanzees and humans has been characterized as *fission-fusion* (Aureli et al., 2008), just like the slight differences observed between the socio-sexual behavior of *Pan troglodytes* and *Pan paniscus*, there are also important differences in the kind and degree of direct social contact observed among *Homo sapiens* and *Pan troglodytes.* The fission-fusion pattern of chimpanzee societies is substantially more fluid than that of humans: chimpanzee individuals within the same community can be alternatively found interacting within groups composed by different subjects at different points in time and in different locations (Aureli et al., 2008), whereas in humans, as bonds are stronger, they are generally more spatially, and socially stable. This observation, could explain why, compared to humans, most members of a chimpanzee community would show a similar composition of their gut microbiota (i.e., based on an incapacity to detect differences between its strongly interacting composing members). The second aspect of the discrepancy may be based on the fact that, while males remain within the same community, forming and strengthening their bonds with other relatives, females migrate between communities (Robinson and Janson, 1987).

Interesting lines of research relating social structure and composition of microbiota could develop from comparisons between the degree of similarity in the microbiota of humans (grouped, e.g., by pattern of social organization), and different non-human primate species (e.g., across different reproductive systems). If more frequent and direct contact promotes greater similarities in the composition of microbiota between individuals, we would expect that the most heavily bonded species (e.g., pair-bonded species), would show greater similarity to humans with stronger social bonds (e.g., couples), followed by the results of non-human primates across distinct reproductive patterns and humans in different types of groups. In this regard, Fincher et al. (2008), provide an important study suggesting an association between human group-level cohesion and pathogenicity. In this study, even after controlling for other confounding variables, human groups with a higher historical pathogen prevalence were also those with the strongest evidence of collectivism-prone *cultural* values, whereas those with a lower historical pathogen prevalence showed stronger cultural values supporting individualism. Therefore, we suggest that in the context of our proposal, this study provides strong evidence suggesting that, in humans, the more cohesive groups (e.g., indexed by more frequent cultural values favoring collectivism, and thus stronger social bonds between subjects) will show stronger similarities in the microbial communities of their individual members.

### MOUTH-TO-MOUTH INTERACTIONS

Costly Signaling theory suggests that a possible function of seemingly “pointless” behaviors or traits, may be the conveyance of honest information that can benefit different interactants; both signalers and observers (Smith and Bird, 2005). In this context, direct means of microbial transmission have higher probabilities of involving potentially costly behaviors that could, nevertheless, provide valuable information to interactants, for example, in the case of mouth-to-mouth kissing (Hughes et al., 2007). In turn, these considerations suggest that the above accounts of the possible function of kissing may be reduced to a single one, in the form of kissing as a social signal or a means of communication. Here, communication may be defined as an action that alters the probability pattern of the behavior of another organism in a way adaptive to either one or both interactants (Wilson, 2000). Thus, either in the form of reconciliation, as a derivative of food sharing, and as a test of the quality of a relationship, kissing can be interpreted as a behavioral signal aimed at increasing the probability of future cooperation. In turn, in the case of the exchange of microbiota via mouth-to-mouth contact, or as a test for the risk of illness, kissing would represent an exchange of potentially costly information (*sensu* Smith and Bird, 2005), again, relating it to communication. The behavior may also be used by group members as an index of the strength of the relationship between two individuals: a group-oriented signal based upon which they can adjust their responses toward the individuals performing it. In the end, perhaps only cross-cultural research investigating the effects of kiss frequency relative to the composition of gut microbiota (to the best of our knowledge, not yet attempted), could bring significant light unto the matter. A comparative approach could focus in the sociosexual behavior of *Pan paniscus*, testing whether partners more often engaged in ventro-ventral copulation, mouth-to-mouth contact, and/or face-to-face interactions, compared to those engaging in other kinds of affiliative behavior, show greater similarities in their gut microbiota.

We cannot be blind to another alternative that may, nevertheless, still lend support to the hypothesis of an association between similar microbial communities and strong social bonds between individuals. Its main distinction lying in the suggestion that sociality does not represent the primary “medium” through which microbial communities across individuals are transmitted or homogenized. From this perspective, instead, given the beneficial effect of focused social contact upon glucocorticoid production (Crockford et al., 2008; Wittig et al., 2008), close social interaction will have a reducing effect on glucocorticoid production, which in turn will improve individual’s capacity for sustaining an effective immune response. That “improved” resistance (e.g., byproduct of intense social support), would allow subjects to remain in close proximity without turning increasingly susceptible to the pathogens transmitted by the social-partner. Again, this possibility would still result in similarities in the microbial communities of closely bonded individuals. Further research should be focused into determining the effects of impairing microbial transmission between subjects while still allowing normal social interactions. For example, this suggestion could be approached by creating social groups where all but one of its members are kept under germ-free conditions by means of antibiotics and then evaluating the survival and reproductive success of the “non-germ free” individuals across groups. While such research design may be difficult, ethically and pragmatically, it could provide a way of discriminating between the alternatives at hand.

## AUTHOR CONTRIBUTIONS

Augusto J. Montiel-Castro, conceived the main premises and relationships that are the focus of this review; wrote the largest portion of the paper; identified the theoretical relevance of the paper; edited the final version of the paper; designed **Figures 2** and **3**. Rina M. González-Cervantes, conceived some of the relationships that are discussed in the review; helped in the theoretical analysis of the premises of the review; wrote one important subsection of the paper; Gabriela Bravo-Ruiseco, wrote specific portions of the review; helped in the suggestion of specific relationships reviewed. Gustavo Pacheco-López, conceived the main premises and relationships that are the focus of this review; identified the theoretical relevance of the paper; designed the structure of the paper; wrote a large portion of the paper; edited the final version of the paper; designed **Figure 1**.

## Conflict of Interest Statement

The authors declare that the research was conducted in the absence of any commercial or financial relationships that could be construed as a potential conflict of interest.
